# Minimally Invasive Midvastus versus Standard Parapatellar Approach in Total Knee Arthroplasty: A Meta-Analysis of Randomized Controlled Trials

**DOI:** 10.1371/journal.pone.0095311

**Published:** 2014-05-20

**Authors:** San-Zhong Xu, Xiang-Jin Lin, Xiang Tong, Xuan-Wei Wang

**Affiliations:** Department of Orthopaedics, First Affiliated Hospital, College of Medicine, Zhejiang University, Hangzhou City, Zhejiang Province, China; University of Michigan, United States of America

## Abstract

**Objective:**

Minimally invasive midvastus approach (mini-midvastus) has been widely used in total knee arthroplasty (TKA). However, the clinical effects still remains controversial. This meta-analysis was based on randomized controlled trials (RCTs) aiming to quantitatively analyze the clinical efficacy of mini-midvastus versus standard parapatellar approach in TKA.

**Methods:**

This meta-analysis was performed according to the PRISMA guidelines. A literature search for the eligible RCTs was carried out in the databases of PubMed, the Cochrane library, EMBASE and Web of Science. Two independent reviewers independently completed the study selection, data extraction, and the assessment of methodological quality. Meta-analysis was conducted by the RevMan 5.2 software.

**Results:**

A total of 18 RCTs (937 patients with 1093 TKAs) published from 2007 to 2013 were included. The meta-analysis suggested that the mini-midvastus approach significantly improved knee range of motion (ROM) and decreased visual analog score (VAS) at postoperative 1–2 weeks (*p*<0.05), and there were no statistical differences in terms of knee society score (KSS) (6 weeks to 1 year), VAS (6 weeks to 6 months), ROM (6 weeks to 6 months), lateral retinacular release, blood loss, straight leg raise, hospital stay and postoperative complications between the mini-midvastus and standard parapatellar approach (*p*>0.05). However, the operative time was significantly longer when performing the mini-midvastus group than the parapartellar approach (*p*<0.05).

**Conclusion:**

This meta-analysis found that compared with the standard parapatellar approach, the mini-midvastus approach had early advantages in the VAS and ROM, but had the disadvantage in the operative time.

**Level of Evidence:**

Therapeutic study Level I.

## Introduction

Minimally invasive surgery (MIS) in total knee arthroplasty (TKA) has been a safe and effective technique for patients with end-stage arthritis [1]. It was originally defined as a small surgical incision less than 14 cm during TKA [2]. However, the current view tends to describe it as a technique which minimizes the disruption of muscle, soft tissue and blood supply during operation. The previous studies have confirmed the MIS technique was associated with less pain, earlier recovery and better quadriceps function than the conventional TKA [2]–[4].

In MIS TKA, subvastus, midvastus and quads-sparing approaches are the most commonly alternatives to standard parapatellar approach [5], [6]. Subvastus and quads-sparing approaches preserved the knee extensor mechanism, and thus were regarded as more minimally invasive than the parapatrllar approach. However, the small surgical field and the increasing operative difficulty limit the popularity theses two approach [7], [8]. As a compromise of these approaches, mini-midvastus approach was introduced as it minimized the vascular and muscular disruption of knee and provided a relatively better operative exposure [6], [9]. Therefore, mini-midvastus approach has probably been the most popular approach in MIS TKA [10].

Currently, numerous well-designed studies have compared the clinical results of mini-midvastus with medial parapatellar approach. However, the conclusions among studies are still controversial. Some studies found no differences between mini-midvastus and parapatellar approaches [5], [11]–[13], whereas others supported the mini-midvastus [14], [15] or standard parapatellar approach [11], [16], [17]. Therefore, we designed this meta-analysis to quantitatively compare the clinical efficacy and safety of mini-midvastus and parapatellar approach in TKA.

## Materials and Methods

Our meta-analysis was strictly conducted according to PRISMA(Preferred Reporting Items for Systematic Reviews and Meta-Analyses) statement – a reporting guideline for meta-analysis [18].

### Inclusion criteria for this meta-analysis

To improve the level of evidence, this study only included published randomized controlled trials (RCTs), but retrospective study, quasi- or non-RCTs were not considered for inclusion. The patients participated in RCT must be adult patients who underwent the primary TKA. All the patients should be divided into two groups: one performed the mini-midvastus approach, and the other performed the standard parapatellar approach. All the parameters of patients such as patient number, age and body mass index (BMI) should be comparable in both groups. The following outcomes were extracted for meta-analysis: (1). Primary outcomes: Knee Society Score (KSS) and Visual analog score (VAS); (2). Secondary outcomes: knee range of motion (ROM), operative time, lateral retinacular release, blood loss, straight leg raise, hospital stay and postoperative complications (total complications, deep vein thrombosis and wound infection).

### Search strategies for identification of studies

A systematic literature search was conducted in PubMed (1950-October, 2013), EMBASE (1974-October, 2013), Cochrane Library (issue 9, 2013) and Web of Science (SCI) (1980- October, 2013). The following search strategies were used in the search: #1. Arthroplasty, Replacement, Knee [Mesh] OR knee arthroplasty OR knee replacement; #2. parapatellar OR standard OR conventional; #3. midvastus OR mini-midvastus OR vastus splitting; #4. #1 AND #2 AND #3. Furthermore, the references lists of included studies and Google scholar were also searched. All the processes were performed by two blinded authors.

### Data extraction and quality assessment

Data was extracted using a pre-designed sheet. The quality assessment of the included studies was evaluated by the Tool recommended by the Cochrane Collaboration [19]. The items, including randomization; allocation concealment; blinding of participants; blinding of outcome assessors; incomplete outcome data; selective reporting; and other bias, were assessed by “Yes”, “No” or “Unclear” by two independent authors. Disagreement was resolved by discussion among authors.

### Statistical analysis

The dichotomous outcomes (lateral retinacular release and postoperative complications) were analyzed with odds ratios (OR) and 95% confidence interval (95% CI). The continuous outcomes (KSS, VAS, ROM, SLR, operative time, blood loss and hospital stay) were analyzed using mean difference (MD) and 95% CI. Statistical heterogeneity were tested with the χ^2^ test and *I^2^* statistic: *I^2^*>50% meaning significant heterogeneity, and *I^2^*≤50% meaning no significant heterogeneity [20]. When heterogeneity was not significant, a fixed effect model was used for meta-anlysis, otherwises a random-effect model was used. Subgroup analysis was conducted in the different types of complications and outcomes at different time points. Meta-analysis was done using the software of Review Manager 5.2.

## Results

### Literature search

**Figure 1:**
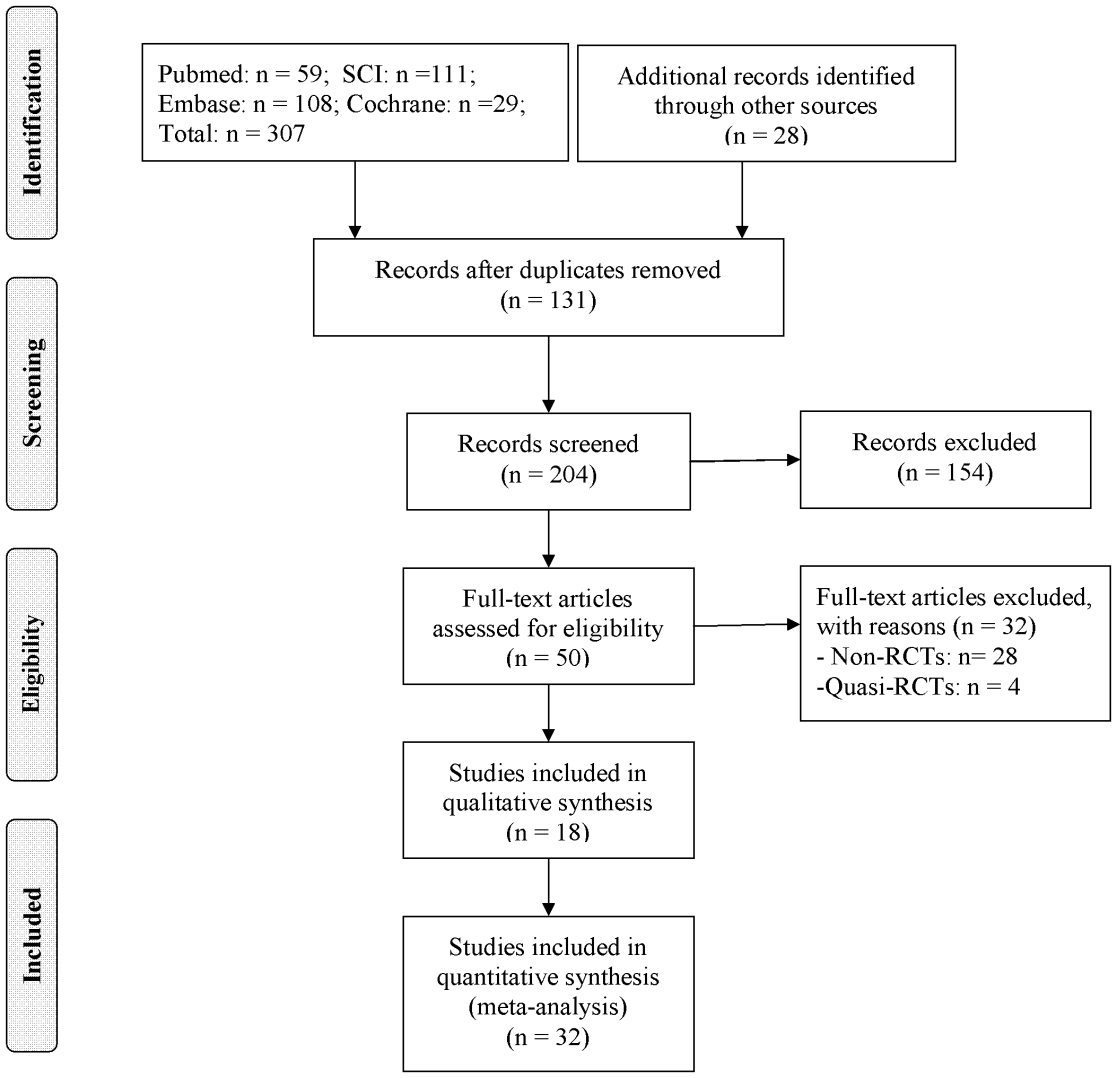
Flow chart of literature screening.

Figure 1 showed the flow chart of the literature search. The initial search found 307 potentially relevant citations from PubMed, EMBASE, Cochrane Library and Web of Science. After carefully screening the title, abstract and full text, 18 RCTs were finally included [5], [11]–[17], [21]–[30].

### The characteristics of included RCTs

Table 1 summarized the basic characteristics of included studies. All the studies were published in 7 years (2007–2013) recently. There were a total of 937 patients (female: 70.2%, male: 29.8%) with 1093 TKAs in the included 18 RCTs. All the included RCTs were small trials with patients' number ranging from 20 to 100. The mean age of the included patients ranged from 62.6 to 71.5 years, BMI 24.4 to 34.8 kg/m^2^, and the follow-up duration 3 months to 3 years. Both groups were well matched in patient number, age, BMI and preoperative knee function and flexion.

**Table 1:** Characteristics of included studies.

Study-year	Year	Country	Group	Age	BMI	Patients	Male	Female	Total TKA	Follow-up
Chin	2007	Singapore	Mini-MV	67.4 (56–80)	28.50(22.1–40.0)	30	6	24	30	3 months
			MP	69.0 (57–80)	27.5 (18.6–34.2)	30	3	27	30	
Cho	2013	Korea	Mini-MV	65.5±5.1	29.1±3.9	33	1	32	33	1 year
			MP	67.0±5.7	28.0±4.1	33	2	31	33	
Dalury	2008	USA	Mini-MV	67 (53–86)	30 (23.6–39)	20	6	14	20	3 months
			MP						20	
Fu	2008	China	Mini-MV	67 (53–86)	30 (23.6–39)	34	7	27	34	3 months
			MP						34	
Guy	2012	UK	Mini-MV	71.2	28.2±3.0	40	38	42	40	1 year
			MP	69.1	28.9±3.8	40			40	
Heekin	2013	USA	Mini-MV	65.13±6.49	30.98±5.44	40	26	14	40	2 years
			Mini-MP						40	
Hernandez-Vaquero	2010	Spain	Mini-MV	70.8±5.9	32.1±6	26	5	21	26	6 months
			MP	70.5±6.9	30.8±3.3	36	6	30	36	
Juosponis	2009	Lithuania	Mini-MV	72±5.5	27.95±3.2	35	5	30	35	3 months
			MP	71.4±5.04	29.08±2.7	35	5	30	35	
Karachalios	2008	Greece	Mini-MV	71.1 (52–78)	32 (27–35)	50	19	31	50	3 years
			MP	70.8 (54–77)	31.5 (28–35)	50	15	35	50	
Karpman	2009	USA	Mini-MV	74±7.7	30±7.3	20	7	13	20	6 months
			MP	73±5.1	29±4.6	19	9	10	19	
Kim	2011	Korea	Mini-MV	67±6	27.1±3	23	0	23	23	1 year
			MP	68±7	28.4±5	22	0	22	22	
Kolisek	2007	USA	Mini-MV	67 (48–84)	32 (19–49)	40	29	11	40	3 months
			MP	70 (54–79)	30 (20–40)	40	24	15	40	
Lee	2011	Korea	Mini-MV	67 (54–73)	27.5 (23.1–33.8)	30	2	28	30	6 months
			MP						30	
Nestor	2010	USA	Mini-MV	66.7±9.6	29.6±5.6	27	9	18	27	6 months
			MP						27	
Pongcharoen	2013	Thailand	Mini-MV	67±4.9	27±2.4	30	5	25	30	1 year
			MP	67±6.0	26±2.3	30	7	23	30	
Varnell	2011	Italy	Mini-MV	75±7	29.5±4.59	20	6	14	20	6 months
			Mini-SV	71±6	30.96±6.16	18	11	7	20	
Walter	2007	USA	Mini-MV	71.5 (53–85)	31.8 (26.2–43.9)	25	6	19	25	3 months
			MP	66.6 (50–80)	34.8 (22.3–59.4)	19	7	12	19	
Zhang	2013	China	Mini-MV	62.6 (48–70)	24.8 (19.6–29.4)	45	14	31	45	6 months
			MP	63.7 (47–74)	24.4(19.2–28.6)	44	14	30	44	

*.Mini-MV =  mini-midvastus, MP =  medial parapatellar, NR =  not report

### Methodological quality assessment

The methodological quality of the included studies was shown in Table 2. All the studies reported that the included participates were randomly assigned to two groups, but four [11], [14], [21], [27] did not mentioned the method of randomization. The method of blinding was performed in 12 of 18 RCTs (66.7%), but allocation concealment was in 5 studies (27.8%).

**Table 2:** Risk of bias in included studies.

Study	Random Sequence Generation	Allocation concealment	Blinding of participants	Blinding of outcome assessment	Incomplete Outcome data	Selective reporting	Other bias
Zhang 2013	Yes (Not reported)	Unclear	Unclear	Unclear	Yes	Unclear	Unclear
Pongcharoen 2013	Yes (Computer-generated numbers)	Unclear	Unclear	Unclear	Yes	Unclear	Unclear
Heekin 2013	Yes (Randomization table)	Unclear	Yes	Unclear	Yes	Unclear	Unclear
Cho 2013	Yes (Not reported)	Unclear	Unclear	Unclear	Yes	Unclear	Unclear
Guy 2012	Yes (Randomization table)	Yes (Sealed envelope)	Unclear	Yes	Yes	Unclear	Unclear
Varnell 2011	Yes (Not reported)	Unclear	Unclear	Unclear	Yes	Unclear	Unclear
Lee 2011	Yes (Computer-generated numbers)	Yes (Sealed envelope)	Unclear	Yes	Yes	Unclear	Unclear
Kim 2011	Yes (Randomization table)	Yes (Sealed envelope)	Yes	Yes	Yes	Unclear	Unclear
Hernandez-Vaquero2010	Yes (Randomization table)	Unclear	No	Yes	Unclear	Unclear	Unclear
Nestor 2010	Yes (Randomization table)	Unclear	Yes	Yes	Yes	Unclear	Unclear
Karpman 2009	Yes (Computer-generated numbers)	Unclear	Yes	Yes	Yes	Unclear	Unclear
Juosponis 2009	Yes (Randomization table)	Yes (Sealed envelope)	Unclear	Unclear	Yes	Unclear	Unclear
Dalury 2008	Yes (Randomization table)	Unclear	Unclear	Yes	Yes	Unclear	Unclear
Karachalios 2008	Yes (Computer-generated numbers)	Yes (Sealed envelope)	Unclear	Yes	Yes	Unclear	Unclear
Fu 2008	Yes (Not reported)	Unclear	Yes	Yes	Yes	Unclear	Unclear
Walter 2007	Yes (Randomization table)	Yes (Sealed envelope)	Unclear	Yes	Yes	Unclear	Unclear
Kolisek 2007	Yes (Randomization table)	Yes (Sealed envelope)	Unclear	Unclear	Yes	Unclear	Unclear
Chin 2007	Yes (Randomization table)	Yes (Sealed envelope)	Yes	Yes	Yes	Unclear	Unclear

### Results of meta-analysis

Table 3 showed the results of meta-analysis.

**Table 3:** Meta-analysis of nini-midvastus versus medial parapatellar approach.

Results	TKAs		Included studies	MD or OR (95% CI); p value	Heterogeneity
	MV	MP			
**KSS 6 weeks**	168	168	5 [11], [15], [20], [26], [29]	5.15 (−3.36, 13.66); *p* = 0.24	*I^2^* = 89%
**KSS 3 months**	168	168	5 [11], [15], [20], [26], [29]	0.55 (−1.41, 2.50); *p* = 0.59	*I^2^* = 0%
**KSS 6 months**	73	73	2 [11], [20]	−2.15 (−5.92, 1.62); *p* = 0.26	*I^2^* = 30%
**KSS 1 year**	103	103	3 [11], [19], [20]	0.66 (−0.68, 1.99); *p* = 0.33	*I^2^* = 26%
**VAS 3 days**	91	91	3 [22], [23], [27]	−0.27 (−0.91, 0.37); *p* = 0.42	*I^2^* = 80%
**VAS 1**–**2 weeks**	91	91	3 [22], [23], [27]	−0.20 (−0.29, 0.11); *p*<0.01	*I^2^* = 0%
**VAS 6 weeks**	87	87	3 [19], [22], [23]	−0.12 (−0.32, 0.07); *p* = 0.22	*I^2^* = 0%
**VAS 3 months**	87	87	3 [19], [22], [23]	−0.02 (−0.21, 017); *p* = 0.82	*I^2^* = 0%
**VAS 6 months**	86	96	3 [19], [22], [24]	−0.02 (−0.20, 015); *p* = 0.80	*I^2^* = 0%
**ROM 1**–**2 week**	146	145	5 [22], [23], [25]–[27]	7.45 (3.26, 11.64); *p*<0.05	*I^2^* = 26%
**ROM 6 weeks**	272	268	8 [15], [20], [22], [23], [26], [27], [33], [34]	2.12 (−2.42, 6.66); *p* = 0.36	*I^2^* = 86%
**ROM 3 months**	151	151	5 [15], [20], [22], [23], [27]	0.72 (−1.20, 2.65); *p* = 0.46	*I^2^* = 0%
**ROM 6 months**	66	76	2 [20], [24]	1.03 (−1.77, 3.83); *p* = 0.47	*I^2^* = 0%
**Operative time**	255	264	8 [14], [19], [22], [24]–[27], [30]	11.64(5.50, 17.78); *p*<0.05	*I^2^* = 93%
**Lateral retinacular release**	329	318	7 [13], [15], [20], [27], [28]	0.67 (0.41, 1.11); p = 0.12	*I^2^* = 0%
**Blood loss**	268	266	8 [5], [11], [13], [19], [22], [25], [29], [30]	1.73 (−1.79, 5.25); *p* = 0.33	*I^2^* = 0%
**Straight leg raise**	144	137	4 [13], [27], [28], [34]	0.43 (0.15, 1.27); *p* = 0.13	*I^2^* = 0%
**Hospital stay**	141	144	5 [20], [24], [25], [28], [30]	1.46(−9.03, 11.94); *p* = 0.79	*I^2^* = 53%
**Total Complication**	405	413	12 [5], [11], [13], [14], [19], [22]–[25], [27], [29], [30]	0.95 (0.53, 1.71); *p* = 0.88	*I^2^* = 0%
**Wound infection**	430	432	13 [5], [11], [13], [14], [19], [22]–[25], [27]–[30]	1.22(0.52, 2.90); *p* = 0.64	*I^2^* = 0%
**Deep vein thrombosis**	254	253	7 [5], [11], [14], [23], [27], [29], [30]	0.46 (0.13, 1.59); p = 0.22	I^2^ = 0%

### Primary outcomes

#### KSS

Six studies were available for meta-analysis of KSS [12]–[14], [17], [26], [29]. Subgroup analyses found no differences between the min-midvastus and standard groups in postoperative 6 weeks (*p* = 0.24), 3 months (*p* = 0.59), 6 months (*p* = 0.26) and 1 year (*p* = 0.33). The heterogeneity was statistically significant in KSS at 6 week (*I^2^* = 89%). (Table 3)

#### VAS

Five studies were included for meta-analysis of VAS [12], [22]–[24], [27]. Subgroup analyses suggested that the mini-midvastus approach significantly decreased VAS at postoperative 2 weeks (*p*<0.01), and no differences were seen between the two groups at the time points of 3 day (*p* = 0.42), 6 week (*p* = 0.22), 3 month (*p* = 0.82), 6 month (*p* = 0.80). The heterogeneity was statistically significant in VAS at 3 days (*I^2^* = 80%). (Table 3)

### Secondary outcomes

Meta-analysis revealed that the mini-midvastus group showed significantly better ROM at postoperative 2 weeks (*p*<0.05) but required longer operative time (*p*<0.05). There were no differences in lateral retinacular release (*p* = 0.12), blood loss (*p* = 0.33), straight leg raise (*p* = 0.13), hospital stay (*p* = 0.79) and postoperative complications (total postoperative, *p* = 0.88; wound infection, *p* = 0.64; deep vein thrombosis, *p* = 0.22). The significant heterogeneity was found in ROM at 6 weeks (*I^2^* = 86%), operative time (*I^2^* = 93%) and hospital stay (*I^2^* = 53%). (Table 3)

## Discussion

The major finding of this study was that the midvastus approach was superior to the standard parapatellar approach in VAS and ROM in the short term (postoperative 2 weeks). There were no statistical differences between the mini-midvastus and parapatellar approach in terms of KSS (6 weeks to 1 year) VAS (6 weeks to 6 months), ROM (6 weeks to 6 months), lateral retinacular release, blood loss, straight leg raise, hospital stay and postoperative complications. In addition, the midvastus approach was found to be associated with significantly longer operative time in TKA.

An earlier meta-analysis [31] had compared the short-term results of the midvastus with the standard approach. Their results showed that the midvastus approach obtained better postoperative recovery, less lateral release and complication rates than the standard approach. However, there were several obvious limitations related to this published meta-analysis. First, that study only analyzed the short-term outcomes without the analysis of the long-term results; second, the data of meta-analysis was based on not only RCTs but also quasi-RCTs, which might lower the strength of evidence; third, the studies which did not use MIS technique in midvastus approach was also included in meta-anlysis, which might effect the specificity of the mini-midvastus; fourth, the meta-analysis should be updated as a number of well-designed RCTs were recently published [5], [11]–[14], [21], [22]. Compared with the published meta-analysis, we included 7 more RCTs [5], [11]–[14], [21], [22] and excluded 4 RCTs [32]–[35] who did not use MIS in the midvastus group. Therefore, we believe our evidence was stronger on the efficiency of the mini-midvastus approach in TKA.

Our meta-analysis was based on 18 RCTs published from 2007 to 2013. The primary outcomes, VAS and KSS, were the top concerns by the patients undergoing primary TKA. However, we only found that the VAS in the mini-midvastus group was reduced in the early period postoperatively. This results were similar with the previous study of Fu et al [27]. In this randomized controlled study with 68 bilateral TKAs, the VAS score of the midvastus group was significantly decreased in the first week after surgery. With regard to KSS, we did not observe any differences between the mini-midvastus and standard approach up to 1 year postoperatively. This corresponded well with the recent studies [5], [11]. Zhang et al. [11] compared 45 midvastus TKAs with 44 parapatellar TKAs and found no significant difference in KSS during the follow-up period (7 days, 6 weeks, 3 months and 6 months). Guy et al. [5] randomized 80 patients to perform mini-midvastus approach or standard parapatellar approach, and also did not find statistical difference in KSS at intervals up to 1 year.

With regard to the secondary outcomes, the current study found significantly higher ROM in the mini-midvastus group than the standard group in the short term (2 weeks), while no differences were found in the later period (6 weeks to 6 months). In addition, we found the mini-midvastus approach significantly increased operative time. That was understandable as the MIS technique in the mini-midvastus group needed more operative procedure and surgical skills. There were no differences in lateral retinacular release, blood loss, straight leg raise, hospital stay and postoperative complications. These results were mostly in accordance well with the previous meta-analysis [31]. In that meta-analysis, the authors found that the midvastus group significantly decreased lateral release rate compared with the standard group, while our result did not find different between two groups. The possible reason was for a quasi-randomized trial [36] they included, in which, the lateral release rate was significantly higher in the midvastus (1/22) than that in the parapatellar group (13/29). When excluding this quasi-randomized trial, the difference disappeared.

This meta-analysis has some limitations. First, despite 18 RCTs were included, the data for meta-analysis was still insufficient, especially for the primary outcomes. Second, the heterogeneity among studies was significant in the KSS at 6 weeks, VAS at 3 days, ROM at 6 weeks and operative time. Although a random-effect model was applied to incorporate heterogeneity in meta-analysis, the readers still should be cautious with these results. Furthermore, some RCTs included for meta-analysis did not carry out binding method and allocation concealment, which might increase a risk of performance and selection bias.

Additionally, there are several strengths in this present meta-analysis. First, the evidence is based on the meta-analysis of RCTs, which is the highest level of evidence (Level I). Second, in order to ensure the accuracy of data, a thorough literature search was conducted for the published studies only, and unpublished studies were not included. Third, we compared the clinical outcomes in the long-term period by subgroup analysis, which was very important to evaluate the safety of a new technique. Fourth, we excluded the studies without using MIS in the midvastus approach, minimized the bias from the differences in the operative procedures, and specially focused on efficacy of the mini-midvastus versus standard approach.

## Conclusions

Based on the meta-analysis of RCTs, we conclude that the mini-midvastus approach is associated with the short-term advantages in the VAS and ROM, but has significantly longer operative time compared with the standard parapatellar approach. There are no statistically differences in KSS lateral retinacular release, blood loss, straight leg raise, hospital stay and postoperative complications between groups.

## Supporting Information

Checklist S1 **PRISMA Checklist.**
(DOC)
